# Clinical predictors of syringomyelia in Cavalier King Charles Spaniels with chiari-like malformation based on owners’ observations

**DOI:** 10.1186/s13028-024-00725-1

**Published:** 2024-02-08

**Authors:** Tenna Remler Pedersen, Maiken Bayer Thode Bach, Camilla Løkke Stougaard, Hanne Gredal, Clare Rusbridge, Nanna Brix Finnerup, Mette Berendt

**Affiliations:** 1 Department of Clinical Veterinary Sciences, Faculty of Health and Medical Sciences, University of Copenhagen, Dyrlaegevej 16, C DK-1870 Frederiksberg, Denmark; 2 Department of Clinical Sciences, School of Veterinary Medicine, University of Surrey, Daphne Jackson Rd, GU2 7AL Guildford, Great Britain; 3 The Danish Pain Research Center, Department of Clinical Medicine, Aarhus University, Palle Juul-Jensens Boulevard 165, 8200 Aarhus N, Denmark

**Keywords:** Chronic pain, Dog, Neuropathic pain, Phantom scratching, Predictive value, Syringohydromyelia

## Abstract

**Background:**

Syringomyelia (SM) is a prevalent inherited developmental condition in Cavalier King Charles Spaniels (CKCSs) with Chiari-like malformation (CM), accompanied by a variety of clinical manifestations, including signs of neuropathic pain. Magnetic resonance imaging (MRI) is the gold standard in SM diagnosis. However, it is desirable to establish clinical predictors that can identify CKCSs with a large clinical syrinx that needs treatment, as some owners cannot afford or lack access to MRI. The aims of the study were to investigate owner-reported clinical signs of SM and clinical predictors of a large clinical syrinx, using predictive values of significant signs, individually and in combinations. Eighty-nine CKCSs participated in this retrospective study. Based on MRI diagnosis, dogs were distributed into three groups: CM without syrinx or with a maximum transverse width < 2 mm (*n* = 13), CM with small syrinx 2.00-3.99 mm (*n* = 26) and CM with large syrinx ≥4 mm (*n* = 50). A structured investigator-owner interview using a standardized questionnaire was used to collect data regarding clinical signs of CM and SM. The statistical tests Pearson’s chi-square, Fisher’s Exact and Spearman’s rank order were used to assess the difference in owner-reported signs between groups. For signs with significant differences, positive and negative predictive values (PPV and NPV) were calculated.

**Results:**

Following clinical signs were reported significantly more frequent in dogs with a large syrinx: phantom scratching, bilateral scratching of the neck or shoulder, aversion when that area is touched, or exacerbation of clinical signs when the dog is emotionally aroused. Each individual sign had a high PPV, indicative of a large clinical syrinx. The PPV increased further when the signs phantom scratching, aversion to touch to the head, neck or shoulder, and a preferred head posture during sleep were present in combination.

**Conclusions:**

Specific clinical signs can be used individually and in combination as clinical predictors of a large clinical syrinx in CKCSs with CM and SM. General practitioners can utilize this information to identify CKCSs with a large syrinx to initiate necessary treatment. This is particularly useful in cases where access to or affordability of an MRI diagnosis is limited.

**Supplementary Information:**

The online version contains supplementary material available at 10.1186/s13028-024-00725-1.

## Background

Syringomyelia (SM) is a prevalent and inherited developmental neurological condition in Cavalier King Charles Spaniels (CKCSs) and other brachycephalic toy breed dogs [1–3]. SM is characterized by the development of one or more fluid-filled cavities within the spinal cord parenchyma documented clinically by MRI or on post-mortem histopathology [4–6]. In the CKCS, SM co-occurs with Chiari-like malformation (CM), a complex developmental condition of the skull and craniocervical vertebrae, characterized by a conformational change and overcrowding of the brain and cervical spinal cord, particularly at the craniospinal junction [7, 8]. In the CKCS, decades of breeding selection towards a distinctive head conformation has led to a high number of individuals with CM and SM [8, 9]. Chiari-like malformation, as defined by the British Veterinary Association/Kennel Club Health Scheme, is ubiquitous in the CKCS [6]. The prevalence of SM has been found to range from 25 to 70% in CKCSs with CM, with an age-increase of 1.27–1.3 in odds ratio per year [10, 11]. Humans also suffer from a similar condition, Chiari malformation type I, with concurrent SM occurring in up to 85% of all patients [12–14]. Chiari-like malformation in CKCSs is comparable to Chiari malformation type I associated with complex craniosynostosis [7]. In both dogs and humans, some individuals have no clinical signs of CM and/or SM despite pathological changes on magnetic resonance imaging (MRI), whereas, in others, CM and/or SM are accompanied by unpleasant clinical manifestations, with pain being one of the most prominent [12, 13, 15, 16]. Both CM severity and increasing syrinx diameter influence the risk of developing clinical signs [17–19].

In humans, CM and SM classification has been related to the clinical phenotype (symptomatology) and natural history of the disease [14, 15]. This classification distinguishes between major neurological symptoms that the patient can report and major neurological signs that can be assessed clinically [15, 16]. The pain phenomenon experienced in humans with SM is most commonly at-level neuropathic pain [20, 21]. This is characterized by an ongoing burning, pricking, squeezing or paroxysmal pain often associated with evoked pain, typically with pain evoked by light touch, e.g. from clothes or contact with cold temperature [21]. Patients with CM/SM also often describe headache, which is often worse with coughing and sneezing (Valsalva maneuvers), and neck pain [15, 16, 22, 23]. However, only one veterinary review has described the possibility of migraine-like pain in a dog with episodic pain behavior [24]. Assessing signs of SM in dogs including pain relies on a combination of the owners’ observations and a professional clinical examination [25]. Recently, it has been reported that certain clinical signs in the CKCS, such as spinal pain, scratching the back of the head or ears, sleep disruption or aversion against being touched on the head or neck, may be linked to CM rather than to SM [18, 19]. This is interesting, and the classification of signs of CM and SM in the CKCSs is worth exploring in more detail, as it helps to systematize knowledge as we advance the understanding of the pathophysiology underlying these conditions. The aim of the present study was to investigate owner-reported clinical signs of SM and to investigate if certain signs were more likely to indicate a large versus a small syrinx in CKCSs with CM. The study also investigated the predictive values of significant clinical signs and combinations of such signs with the purpose of establishing clinical predictors of a large clinically relevant syrinx.

## Methods

### Study design and description of study population

The study was conducted in 2022 and used retrospective information as well as data collected at the time of the study. The study population consisted of 89 CKCSs investigated with MRI at the University Hospital for Companion Animals, University of Copenhagen, between 2007 and 2014: 76 dogs were diagnosed with CM and SM and 13 dogs with CM and no SM. Sixty-five dogs were referred to MRI after a clinical and neurological evaluation at the University Hospital’s Neurology Specialist Clinic and 24 dogs were referred directly to MRI at the University Hospital’s Imaging Department from private practice.

All dogs had a hospital record, including a full ID of the dog and owner, and an MRI confirmed diagnosis of CM with or without SM established by a diplomat of the European College of Veterinary Diagnostic Imaging (ECVDI) or an ECVDI resident under diplomat supervision at the University Hospital’s Imaging Department. All owners participated in an investigator-owner interview using a standardized questionnaire targeting possible clinical signs of CM and SM.

### Magnetic resonance imaging

Dogs were scanned with a 0.2 Esaote Vet MR; Esaote, Genoa, Italy. For each dog, a minimum of the caudal neurocranium and the first five cervical segments of the spinal cord region were included in the scan. The scanning protocol, as a minimum, included T1-weighted and T2-weighted sequences in sagittal and transverse orientation. For the present study, the first author (TRP, PhD student in veterinary neurology), in 2022, reevaluated all MRI scans and additionally evaluated the grade of CM and performed detailed measurements of syrinx diameters.

CM was categorized according to the British Veterinary Association/Kennel Club scheme with ‘CM grade 0’ (No CM), ‘CM grade 1’ (Cerebellum indented) or ‘CM grade 2’ (Cerebellum impacted into or herniated through foramen magnum).

If a syrinx was present, the syrinx diameter was measured as the maximum transverse diameter using T1-weighted cervical transverse scans to avoid the risk of overestimating the diameter that may occur if using T2-weighted scans [26]. A syrinx was defined by a transverse diameter of at least two mm. The shape of the syrinx was classified as central, right- or left-lateralized or bilateral. A syrinx was defined as lateralized if extending into one spinal cord dorsal horn only and bilateral if extending into both dorsal horns. A central syrinx was circular and extended equally in all directions. SM was further subcategorized as either a ‘small syrinx 2.00-3.99 mm’ (syrinx diameter) or a ‘large syrinx ≥4 mm’.

For the evaluation of MRI scans, dogs were distributed into three groups: (1) dogs with CM and no SM (or with a maximum transverse width < 2 mm), (2) dogs with CM and small syrinx (SM 2.00-3.99 mm) and (3) dogs with CM and large syrinx (SM ≥4 mm) [18].

Furthermore, the presence of effusive otitis media (OME) was documented, including information on whether OME was bilateral or unilateral. In cases of unilateral OME, the specific side (right or left) was also recorded.

### Questionnaire data

The owners of all dogs participated in a structured interview targeting clinical signs of CM-SM. Investigator-owner interviews were performed in the clinic face to face (*n* = 35) or by telephone (*n* = 54) in 2014. In order to secure a uniform interview procedure, all interviews were performed by only two investigators (MBTB and CLS) using a standardized questionnaire (Supplementary material, Table S1). Informed consent was obtained from the owners before the interview. The owners were asked to answer the questions based on their recollection of the dog’s clinical signs at the time of the MRI scan. The questionnaire consisted of 14 dichotomous questions (yes or no), out of which 10 addressed clinical signs related to CM-SM (question three, five to nine and twelve to fifteen). Furthermore, the questionnaire contained one nominal question regarding lateralization of scratching (question four) and five ordinal questions (question two, ten, eleven, sixteen and seventeen) with a grading of the clinical signs (never (0), occasionally (1), often (2) or always (3)) (Supplementary material, Table S1).

To separate scratching with skin contact (question two) from phantom scratching (with no skin contact), the owners were asked whether their dog would ever scratch towards neck or shoulder without the paw touching the skin (question five). Phantom scratching was defined as a rhythmic scratching action towards neck or shoulder region, but not contacting the skin, together with a curvature of the body and neck towards the foot [27].

Knowing that headaches are associated with CM-SM in humans [15, 16, 22, 23] we inquired, for exploratory purposes, if the owners suspected their dogs to suffer from headaches; if so, they were encouraged to elaborate on what made them think so (Supplementary Table S1, question 12).

All questionnaire data were analyzed in 2022 by two investigators (TRP and MBTB). Methods used for data analysis are described in the statistics section.

Some of the dogs participating in the present study also participated in a study investigating myxomatous mitral valve disease severity in CKCSs with and without SM (unpublished data). The data used for the present study have not been analyzed or published previously.

### Statistical analysis

All statistical analyses were performed with the software RStudio version 1.4.1717, Stata version 18 and IBM SPSS Statistics version 28. The statistical significance level was set at *P* < 0.05.

The study presented descriptive MRI data, which included information about the presence of CM and SM, as well as the diameter and distribution of the syrinx (whether it was central, right-sided, left-sided, or bilateral). The data was shown as medians with interquartile ranges or as proportions. Additionally, the average transverse syrinx diameter was represented by providing both the minimum and maximum values. For the questionnaire data, percentages were used along with the total numbers within groups (CM and no SM, CM and SM 2.00-3.99 mm, and CM and SM ≥4 mm).

To assess differences in the distribution of categorical variables between groups, Pearson’s chi-square test was employed for dichotomous and nominal categorical variables. Spearman’s rank-order coefficient was used for ordinal variables. In cases where there were fewer than five instances, Fisher’s Exact test was applied.

Positive and negative predictive values (PPVs and NPVs, respectively) were calculated for statistically significant clinical signs associated with having a syrinx ≥4 mm. Furthermore, PPV and NPV were calculated for significant signs of scratching in combination with other significant clinical signs associated with having a syrinx ≥4 mm.

Finally, the association between syrinx lateralization upon MRI and clinical unilateral scratching of the neck or shoulder was investigated using logistic regression to calculate risk ratios of dogs with a syrinx and lateralized scratching.

**Table 1:** Syrinx maximum transverse diameter and lateralization

		Syrinx (*n* = 76)	Syrinx 2.00-3.99 mm (*n* = 26)	Syrinx ≥4 mm (*n* = 50)
Syrinx maximum transverse diameter (mm)*	Median IQR Min-Max	5.0 3.0-6.9 2.0-9.7	3.0 2.4–3.7 2.0–3.7	6.1 4.0–8.1 4.0–9.7
Syrinx placement**	Central	39 (51.3%)	22 (84.6%)	17 (34.0%)
	Right side	11 (14.5%)	1 (3.8%)	10 (20.0%)
	Left side	18 (23.7%)	2 (7.7%)	16 (32.0%)
	Bilateral	8 (10.5%)	1 (3.8%)	7 (14.0%)

* The diameter was measured at the widest point of the syrinx in transverse sections of MRI scans

** The syrinx lateralization was registered for the widest diameter of the largest syrinx in the cervical region (if more than one syrinx was present). A central syrinx was circular and situated centrally in the spinal cord, whereas a unilateral syrinx was asymmetrical and extended into one dorsal horn only. A bilateral syrinx extended into both dorsal horns

IQR: interquartile ranges; Min: Minimum; Max: Maximum; n: number of dogs

## Results

Clinical data were available for the 65 dogs that were examined at the University Hospital’s Neurology Specialist Clinic. Out of those, 47 dogs had abnormal findings on the clinical and/or neurological examination (Supplementary material, Table S2).

Out of the 89 CKCS participating in the study, 76 (39 females and 37 males) had CM and SM, and 13 (four females and nine males) had CM and no SM. Seventy-five out of 76 dogs with CM and SM had CM grade 2 and one dog was categorized as CM grade (1) All 13 dogs with CM and no SM had CM grade (2) The average diameter of a small syrinx was 3.0 mm (minimum 2.0 mm, maximum 3.7 mm) and 6.1 mm (minimum 4.0 mm, maximum 9.7 mm) for a large syrinx (Table 1). Fifty out of 76 dogs had CM and a large syrinx with a diameter of 4 mm or more, whereas 26 dogs had CM and a small syrinx with a diameter between 2.00 and 3.99 mm. Syrinx diameter and shape (central, lateralized or bilateral) are presented in Table 1. The average age for dogs with CM and a small syrinx (*n* = 26) at the time of MRI was 4.8 years (95% CI [3.9–5.7]), 4.8 years (95% CI [4.2–5.3]) for dogs with CM and a large syrinx (*n* = 50) and 5.3 years (95% CI [4.2–6.2]) for dogs with CM and no SM (*n* = 13). For dogs with CM and no SM, six dogs were older than 5 years and seven dogs were four to five years old at the time of the MRI scan.

At the time of the interview, 34 dogs had died; three dogs with CM and no SM, twelve dogs with CM and small syrinx and 20 dogs with CM and large syrinx. The time from the dogs’ MRI scan to the time of the interview appears from Supplementary Table S3.

Owner-reported clinical signs for the three groups, CM and no SM, CM and small syrinx and CM and large syrinx, are presented in Table 2. The owners did not report any clinical signs for eight out of 13 dogs with CM and no SM, 15 out of 26 dogs with CM and a small syrinx and seven out of 50 dogs with CM and a large syrinx. Dogs with CM and no SM and dogs with CM and small syrinx had fewer clinical signs than dogs with CM and large syrinx (Table 2). For seven clinical signs, there was a statistically significant difference between dogs with CM and a large syrinx compared to dogs with CM and no SM and CM and a small syrinx. The clinical signs, unprovoked scratching (spontaneously induced and with no obvious reason), scratching the neck/shoulder with skin contact, bilateral scratching, phantom scratching, aggravation of scratching during emotional arousal, aversion to touch on head, neck or shoulder and aversion towards wearing a collar or harness, were significantly more frequent in dogs with CM and large syrinx compared to dogs with CM and small syrinx (Table 2). While unprovoked scratching, scratching the neck or shoulder, bilateral scratching, aggravation during emotional arousal and sleeping with a preferred head posture (e.g. elevated) were all reported significantly more frequent in dogs with a CM and a large syrinx than in dogs with CM and no syrinx (Table 2).

**Table 2:** Clinical signs in dogs with no syrinx, small syrinx, and large syrinx

Clinical sign	Answers	CM and no SM (n = 13)	Syrinx 2.00-3.99 mm (n = 26)	Syrinx ≥ 4 mm (n = 50)	*P*
Unprovoked scratching ^Q2^	Sometimes	15.4% (2)	23.1% (6)	20.0% (10)	**< 0.001**
	Often	23.1% (3)	23.1% (6)	54.0% (27)	
	Always	7.7% (1)^a^	0.0% (0)^a^	12.0% (6)^b^	
Scratching neck or shoulder ^Q3^	Yes	23.1% (3)^a^	42.3% (11)^a^	78.0% (39)^b^	**< 0.001**
Unilateral scratching ^Q4^	Unilateral*	0.0% (0)	0.0% (0)	4.1% (2)	**0.015**
	Right side	27.3% (3)	17.4% (4)	30.6% (15)	
	Left side	9.1% (1)	13.0% (3)	18.4% (9)	
	Bilateral	0.0% (0)^a^	13.0% (3)^a^	26.5% (13)^b^	
		*n = 11*	*n = 23*	*n = 46*	
Phantom scratching ^Q5^	Yes	30.8% (4)^a^	23.1% (6)^a^	57.1% (28)^b^ *n = 49*	**0.011**
Aggravation of scratching during emotional arousal ^Q6^	Yes	23.1% (3)^a^	26.9% (7)^a^	59.2% (29)^b^ n = 49	**0.007**
Aversion to touch on head, neck or shoulder ^Q7^	Yes	38.5% (5)^a^	19.2% (5)^a^	58.0% (29)^b^	**0.005**
Signs of neck pain ^Q8^	Yes	38.5% (5)	15.4% (4)	38.0% (19)	0.11
Aversion to wearing collar/harness ^Q9^	Yes	30.8% (4)^b^	3.8% (1)^a^	38.0% (19)^b^	**0.006**
Reduced activity ^Q10^	Sometimes	7.7% (1)	8.0% (2)	12.0% (6)	
	Often	7.7% (1)	20.0% (5)	10.0% (5)	0.67
	Always	7.7% (1)	4.0% (1)	10.0% (5)	
			*n = 25*		
Spontaneous vocalization ^Q11^	Sometimes	30.8% (4)	50.0% (13)	34.0% (17)	
	Often	7.7% (1)	7.7% (2)	22.0% (11)	0.35
	Always	7.7% (1)	3.8% (1)	4.0% (2)	
Signs of headache ^Q12^	Yes	7.7% (1)	26.9% (7)	30.0% (15)	0.26
A preferred head posture during sleep ^Q13^	Yes	0.0% (0)^a^	15.4% (4)^a^	36.0% (18)^b^	**0.011**
Disrupted sleep ^Q14^	Yes	30.8% (4)	26.9% (7)	30.0% (15)	0.95
More nervous/ aggressive ^Q15^	Yes	38.5% (5)	30.8% (8)	30.0% (15)	0.84
Withdrawing from humans ^Q16^	Sometimes	7.7% (1)	7.7% (2)	12.0% (6)	
	Often	15.4% (2)	7.7% (2)	22.0% (11)	0.10
	Always	0.0% (0)	3.8% (1)	4.0% (2)	
Withdrawing from other dogs ^Q17^	Sometimes	30.8% (4)	26.9% (7)	22.0% (11)	
	Often	0.0% (0)	3.8% (1)	26.0% (13)	0.36
	Always	23.1% (3)	19.2% (5)	14.0% (7)	

Data are presented as percentages with total numbers within each group. For questions where the owner could not provide an answer, the dog was excluded from the question. For such questions, the total number of dogs is registered in the table below the specific answer

Within a row, different superscript letters indicate a statistically significant difference between groups based on the post hoc analysis. All the groups denoted with the letter a, are significantly different from the groups denoted with the letter b

* Unilateral was recorded when the owner was sure that the dog had unilateral scratching but did not remember to which side. If the owners were certain that their dog had unilateral scratching of a specific side, this side was registered as right side or left side

CM: Chiari-like malformation; n: total number, Q: question number referring to the questionnaire; SM: syringomyelia

One clinical sign (“Aversion towards wearing a collar/harness”) was significantly less frequently reported in dogs with CM and small syrinx (*P* = 0.006) compared to dogs with a large syrinx and dogs with no syrinx (Table 2). There was, however, no significant difference when comparing dogs with a large syrinx to dogs with no syrinx. Therefore, no further statistical analysis was performed for this clinical sign.

Out of the 76 dogs with CM and SM, 33 had unilateral scratching of the neck or shoulder regardless the size of their syrinx, 15 had bilateral scratching, and seven owners could not recall (Q4, Table 2). Thirteen dogs with a large syrinx had bilateral scratching compared to only three dogs with a small syrinx and no dogs with only CM (Table 2). The logistic regression analysis showed that there was a significant association between syrinx lateralization and ipsilateral scratching of the neck or shoulder (*P* = 0.012). The risk of having a lateralized syrinx was almost twice as high for dogs with ipsilateral scratching compared to dogs with no ipsilateral scratching (relative risk = 1.99). For 23% and 57% of dogs with small syrinx and large syrinx, respectively, the owners reported phantom scratching (Table 2). For dogs with CM and no SM, owners reported that 30% of the dogs had phantom scratching.

Twenty-three owners of dogs, one with CM and no SM, seven with CM and a small syrinx, and 15 with CM and a large syrinx suspected that their dogs suffered from headaches (Q12, Table 2). The owners reported that this concern arose from observations or from intuition (having an in-depth knowledge into their dog’s normal behavior but not being able to specify further). Among observations, abnormal behaviors such as withdrawal behavior, a preference for quiet rooms and episodes with semi-closed eyes were most commonly reported.

The PPV and NPV were calculated for each of the seven significant clinical signs associated with a syrinx of 4 mm or above. All these clinical parameters had a PPV of ≥0.71, with a preferred head posture during sleep having the highest PPV (0.82) (Table 3). This clinical sign also had the lowest NPV (0.52). Dogs with either unprovoked scratching, scratching of neck or shoulder or with phantom scratching had a PPV of 0.73 and an NPV of 0.76, while dogs with unprovoked scratching, scratching of neck or shoulder and phantom scratching in combination had a PPV of 0.75 and an NPV of 0.58 associated with having a large syrinx (≥4 mm). Positive and negative predictive values for dogs with either unprovoked scratching, scratching of neck or shoulder or phantom scratching in combination with the other significant clinical signs (aggravation during emotional arousal, aversion of touch to the head, neck or shoulder and sleeping with a preferred head posture) are presented in Table 4.

No association was found between OME and scratching the neck or shoulder. The same applies to unilateral scratching towards the side of the OME (Supplementary material, Table S4).

**Table 3:** Positive and negative predictive values for having a large syrinx (≥ 4 mm)

Clinical sign	PPV	NPV
Unprovoked scratching ^Q2^		
- Yes/No - Often-Always/ Sometimes-Never	0.71 0.77	0.75 0.63
Scratching neck or shoulder ^Q3^	0.74	0.69
Unilateral scratching ^Q4^ in dogs scratching the neck or shoulder	0.72	-
Bilateral scratching ^Q4^ in dogs scratching the neck or shoulder	0.80	-
Phantom scratching ^Q5^	0.74	0.58
Aggravation of scratching during emotional arousal ^Q6^	0.74	0.59
Aversion to touch on head, neck or shoulder ^Q7^	0.74	0.58
A preferred head posture during sleep ^Q13^	0.82	0.52

NPV: negative predictive value; PPV: positive predictive value; Q: question number referring to the questionnaire; SM: syringomyelia

**Table 4:** Predictive values for combinations of significant clinical signs for a large syrinx (≥ 4 mm)

Unprovoked scratching ^Q2^ OR scratching neck or shoulder ^Q3^ OR phantom scratching ^Q5^	Aggravation of scratching during emotional arousal ^Q6^	Aversion to touch on head, neck or shoulder ^Q7^	A preferred head posture during sleep ^Q13^	PPV	NPV	
X	X			0.74	0.58	
X		X		0.82	0.52	
X			X	0.82	0.52	
X	X	X		0.77	0.53	
X	X		X	0.88	0.51	
X		X	X	0.91	0.49	
X	X	X	X	0.79	0.57	

Positive and negative predictive values for the clinical signs unprovoked scratching ^Q2^ or scratching neck or shoulder ^Q3^ or phantom scratching ^Q5^ in combination with the other significant clinical signs. NPV, negative predictive value; PPV, positive predictive value; Q, question number referring to the questionnaire

## Discussion

This study investigated if certain clinical signs are associated with syrinx size and if specific clinical signs, or combinations of signs, can act as predictors of a large clinically relevant syrinx in CKCSs with syringomyelia.

We found that scratching the neck or shoulder, unprovoked scratching, unilateral scratching, aggravation during emotional arousal and/or a preferred head posture during sleep were all significantly more frequent in dogs with a large syrinx (≥4 mm) compared to dogs with no syrinx and small syrinx. This is in agreement with results of previous studies investigating clinical signs related to SM [18, 28].

To assess the reliability of significant clinical signs to truly detect dogs with CM and a large syrinx, we calculated PPVs and NPVs, assuming that the higher the PPV, the more relevant the clinical marker.

The highest PPV for a single clinical sign was observed in dogs with a large syrinx (*n* = 18) exhibiting a preferred head posture during sleep (PPV = 0.82). However, it is worth noting that this particular clinical sign had the lowest NPV (0.52), meaning that the absence of this clinical sign does not rule out a syrinx. Previous reviews/educational papers have discussed a preferred (commonly elevated) head position during sleep as a sign of posture-induced neuropathic pain arising from CM and SM [29, 30] and it has also been suggested that this head position may be associated with CM rather than SM [19]. None of the CKCSs diagnosed with CM and no SM in the present study displayed an elevated or otherwise unusual head position during sleep, which is in agreement with the results from a previous study [18]. The same study furthermore reported a tendency towards an unusual head position during sleep in dogs with a syrinx ≥ 4 mm, although not statistically significant [18]. Our study found that a significantly larger proportion of dogs with a large syrinx were reported to sleep with an unusual head posture compared to dogs with a small syrinx or no syrinx. The difference in the results between these two studies may be due to the differences in study design. While both studies were retrospective, the former study relied on information coming from medical records, which means that some clinical signs may have been underreported, as the owners were not interviewed with a questionnaire. However, the compiled results from our study and the aforementioned research indicate that an unusual or preferred head posture during sleep may be associated with SM or the combination of SM and CM rather than with CM alone.

The PPV for CM and large syrinx was found to be 76.7% for frequent-to-consistent unprovoked scratching and 74.4% for aversion to being touched on the head, neck or shoulder indicating that these common clinical signs can serve as clinical markers of a large syrinx, as CKCSs displaying these clinical signs are very likely to have a syrinx of 4 mm or greater. Phantom (or fictive) scratching has previously been confirmed as strongly associated with SM and a large syrinx secondary to CM in CKCS [18, 27]. This clinical sign may be induced by light rubbing of the neck or ear region and triggered by emotional arousal, and exercise, and is likely attributed to a maladaptive reflex arc involving the lumbosacral scratching central pattern generator and should not be mistaken for scratching with skin contact [27].

MRI is considered the gold standard diagnostic test for CM and SM but may not be accessible to some owners due to economic or geographic restraints. In such cases, a clinical predictor based on significant cardinal signs would be helpful [31]. In this study, the highest PPV (0.91) was obtained when combining scratching behavior, aversion to being touched on the head, neck or shoulder and a preferred head posture during sleep, indicating that the likelihood of a large clinically relevant syrinx is very high in a CKCS with this combination of clinical signs. Most NPVs were fairly low in our study (Tables 3 and 4), which could indicate a high number of false negative cases. However, as PPV increases with a high prevalence, NPV will automatically decrease [31]. The presence of one or more of the clinical signs reported in this study increases the possibility that the dog may have a large clinically relevant syrinx, while the absence of the clinical signs does not exclude a large syrinx. Some dogs with syrinx (small or large) may be clinically silent or have subtle signs that do not require treatment [32]. The individual signs and the accumulation of clinical signs identified in the present study indicate a clinically relevant large syrinx that needs treatment.

Some of the dogs had dermatological findings on the clinical examination, and thus we cannot exclude that some of the scratching reported by the owners could be due to a dermatological condition. However, the high PPVs in our study indicate a low number of false positive results, suggesting that the clinical signs are highly predictive of a large clinically relevant syrinx.

We found a significant association between syrinx lateralization and ipsilateral scratching. However, we only examined syringes in the cervical spinal cord and SM can also affect the thoracic and lumbar spinal cord [26]. A large thoracic syrinx might influence the clinical signs, such as phantom scratching towards the sternum [18, 26]. In humans, no consistent correlation has been found between syrinx dimension and clinical signs, but neuropathic pain is often located to the same side as the syrinx in case of unilateral placement, similar to our findings [33]. Ipsilateral scratching in dogs may reflect ongoing at-level neuropathic pain in humans [20, 21]. Neuropathic pain is located in areas of sensory loss and sometimes includes evoked pain [20, 21]. In this study, we did not specifically assess sensory loss. However, we observed that dogs with SM often exhibited apparent discomfort when touched. This clinical sign could potentially indicate the presence of evoked neuropathic pain.

In CKCSs with CM and SM, withdrawn behavior marked by a tendency to avoid social interactions has been interpreted as a possible response to pain [19, 34]. A previous study reported a significant association between dogs with CM and SM and anxious or aggressive behavior [35], while another found that a significantly higher proportion of dogs with SM ≥4 mm had a tendency to withdraw from human contact [18]. This present study found no significant difference for neither withdrawal behavior nor aggressive/nervous behavior between dogs with CM and no SM and CM with small or large syrinx. The contradicting results may be explained by the different study and questionnaire designs used for the studies in question. As an example, we used an ordinal 4-point scale to describe the frequency of withdrawn behavior, whereas another study used a semantic 5-point differential-type scale [35]. However, due to study design, it was not possible to differentiate between clinical signs coming from SM and CM in the present study, but it is of interest to discriminate between such signs in future studies.

In the present study we explored the question if dogs, as humans with CM-SM, may suffer from headaches (question 12 in Supplementary Table S1). Quite a high number of owners answered yes based on intuitive belief or observations of abnormal behavior such as withdrawal, a preference for quiet rooms and episodes with semi-closed eyes (which have previously been associated with possible photophobia) [24]. In a recent human classification study of CM and SM, headache was reported in 28% of patients with symptomatic SM and 48% of patients with symptomatic CM and no SM [15]. It is, however, not possible to determine if the signs reported by the owners participating in the present study were actually associated with headache, as such signs may also arise from other pain-related problems associated with CM and SM. At this point, no criteria for objective assessment of potential headaches in dogs have been established in veterinary medicine and only one paper has described a dog with paroxysmal pain episodes including vocalization and fearful behavior suggestive of migraine-like behavior [24]. Just before the onset of the episodes, the dog would appear quiet, retract from social interactions, and hide under furniture. Similar clinical signs were reported by some owners in our study. Although measuring pain experience in animals is difficult, evaluation of pain-like behaviors is increasingly being used in experimental animal models of migraine and headache [36]. Therefore, we consider it relevant to report the owners’ response to question 12 and propose that future attention should be given to further explore, if more subtle behavioral changes in dogs with CM and SM could indicate headache as a hitherto unrecognized welfare problem in some of these dogs.

Earlier research investigating the relationship between owner-reported scratching and pain signs in CKCS with CM and SM showed no association between clinical findings on the neurological examination and presence of syrinx and owner-reported findings [28]. Due to the retrospective nature of our current study, we unfortunately cannot make solid comparisons between these previous findings and our own results.

Previous studies have reported scratching of the neck as a clinical sign potentially linked to OME in CKCSs [37, 38]. In this study, no significant association was found between OME and scratching the neck or shoulder. The studies furthermore mentioned head and neck pain and vocalization as additional clinical signs of OME [37, 38]. It is important to note that none of these studies ruled out the presence of SM and CM, which could have been the underlying cause for the clinical signs reported [37, 38].

We acknowledge that there are certain limitations associated with our study. One is the size of the study population (*n* = 89), and especially the low number of dogs with CM and no SM (*n* = 13), which may have affected the power of the results. The limited spatial resolution of the MRI scanner used in this study did not allow detection of dogs with a possible small central canal dilatation < 2 mm. Consequently, we cannot totally exclude that dogs with such subtle spinal cord findings might have been included in the group of dogs with CM and no SM.

The variable individual time from MRI to the questionnaire interview may have created recall bias and we acknowledge that this is a weakness to the study. Recall bias is, however, most likely to occur in cases where subjects do not find the condition important, and the questions in the questionnaire concerned chronic signs as opposed to single events, which strengthens the owners’ recollection [39]. Furthermore, the owners had previous to 2014, at the time of the MRI scan, reported their dogs’ clinical signs, which makes it less likely that they would forget them. Finally, all questionnaire interviews were performed as oral structured interviews by the same two interviewers who carefully interviewed the owners in the same way, posing questions that were clear and concise. This served to minimize both recall bias and interviewer bias (the risk of influencing the owners’ responses) and thus increased the likelihood of obtaining truly representative data [39].

## Conclusions

The presence of one of the following clinical signs; phantom scratching, bilateral scratching of the neck or shoulder, aversion when that area is touched, or exacerbation of clinical signs when the dog is excited or nervous, each serves as a reliable predictive indicator of CM and a large and clinically relevant syrinx in CKCSs. The positive predictive value is even higher when a combination of phantom scratching, aversion to touch to the head, neck or shoulder, and a preferred head posture during sleep are all present in the same dog.

The information coming from our study is particularly valuable for general practitioners who can use the specific clinical signs, individually or in combination, as a clinical predictor of a large clinically relevant syrinx. This approach can help identify dogs that require treatment, even in situations where owners may not have access to or cannot afford an MRI scan. By recognizing these clinical signs, veterinarians can provide early intervention and appropriate care for affected CKCSs, improving their overall well-being and quality of life.

### Electronic supplementary material

Below is the link to the electronic supplementary material.

Supplementary Material 1

Supplementary Material 2

Supplementary Material 3

Supplementary Material 4

## Data Availability

The datasets used and/or analyzed during the current study are available from the corresponding author upon reasonable request.

## Declarations

### Prior publication

None of the data used in the present study has been published previously.
