# Supplementary material for: Clinical predictors of syringomyelia in Cavalier King Charles Spaniels with chiari-like malformation based on owners’ observations
Source: Acta Vet Scand. 2024 Feb 8;66:5. doi: 10.1186/s13028-024-00725-1 (PMC10851586; doi:10.1186/s13028-024-00725-1)
Supplement: Supplementary file 1 — Supplementary Material 1 [file 13028_2024_725_MOESM1_ESM.docx]

## **Supplementary material**

## **Table S1:** Questionnaire

| Date: |  |  |
| --- | --- | --- |
| Interviewer: |  |  |
| Patient: |  |  |
| Medical record nummer |  |  |
| Owner: |  |  |
| Possible year of death: |  |  |
| Possible cause of death: |  |  |
| *Scale: 0 = Never, 1 = Once, 2 = Often, 3 = Always* | | |
|  |  |  |
| 1. Does your dog show signs of syringomyelia?  *(Syringomyelia is a disease in which a fluid-filled cavity forms in the spinal cord. It is believed to be due to a mismatch between the size of the posterior part of the skull and the brain. The disease can give rise to neurological symptoms that can manifest in different ways.)*  The owner’s observations (if any): Describe if you have observed any signs indicating syringomyelia in your dog. |  | Yes ( ) No ( )  Remarks: |
| 2. Have you experienced that your dog scratches itself for no obvious reason, e.g. sitting down abruptly on walks and starts scratching? |  | Scale (0-3): ( )  If scratching, on a scale from (0-10), how severe is the scratching? |
| 3. If your dog scratches itself, is it directed towards the shoulder and/or neck region?  If “no”, does your dog scratch elsewhere? |  | Yes ( ) No ( ) |
| 4. If your dog scratches itself, is it directed against one side only (right or left) or both sides? |  | Yes ( ) No ( )  Left ( ) Right ( ) Both ( ) |
| 5. Is scratching performed without the paw touching the skin? |  | Yes ( ) No ( ) |
| 6. Do these signs worsen with e.g. barking / agitation/activity/walking on a leash or harness? |  | Yes ( ) No ( )  Remarks: |
| 7. Does your dog show discomfort when you touch the head, neck, or shoulder region? |  | Yes ( ) No ( )  Remarks: |
| 8. Does your dog express signs of neck pain?  *E.g. does the dog walk with her/his head lowered, supporting her/his head on pillows when resting or express signs of pain when changing head position?* |  | Yes ( ) No ( )  Remarks: |
| 9. Does your dog avoid or express signs of discomfort when wearing a collar or harness? |  | Yes ( ) No ( )  Remarks: |
| 10. Have your dog lost interest in/or is less interested in daily walks?  Has this changed over time? If so, can that be related to anything special? |  | Scale (0-3): ( )  Remarks: |
| 11. Does your dog have abrupt yelping or vocalization episodes as being in pain? If yes, is anything in specific triggering such episodes? |  | Scale (0-3): ( )  Remarks: |
| 12. Do you suspect that your dog may suffer from headaches?  If yes, what makes you think so? |  | Yes ( ) No ( )  Remarks: |
| 13. Does your dog prefer to sleep with her/his head in unusual positions, for example, lifted? If yes, please describe this. |  | Yes ( ) No ( )  Remarks: |
| 14. Is your dog’s sleep interrupted? (e.g. does she/he change position often, or are you woken up during the night because your dog is restless and walks around)? |  | Yes ( ) No ( )  Remarks: |
| 15. Do you think your dog appears more nervous or aggressive than before or compared to other dogs in general?  If yes, please describe her/his behavior: |  | Yes ( ) No ( )  Remarks: |
| 16. Have your dog started to withdraw from her/his usual family interactions or hide, making you suspect that something is wrong?  If yes, please describe: |  | Scale (0-3): ( )  Remarks: |
| 17. Does your dog avoid playing with or interacting with other dogs?  If yes, please describe: |  | Scale (0-3): ( )  Remarks: |
| 18. Does your dog receive any medication or supplements?  If yes, please provide the name and dose: |  | Yes ( ) No ( ) |
| 19. Do you have a pedigree for your dog?  If yes, could we get a copy of this? |  | Yes ( ) No ( ) |
| 20. Do you know of any relatives of your dog that have been diagnosed with syringomyelia?  If yes, please describe which relatives: |  | Yes ( ) No ( )  Remarks: |
| Is there anything else you wish to mention? |  | Remarks: |
| May we contact you again if needed?  Contact Information: |  | Yes ( ) No ( )  Phone number:  Email: |
