# Supplementary material for: Clinical predictors of syringomyelia in Cavalier King Charles Spaniels with chiari-like malformation based on owners’ observations
Source: Acta Vet Scand. 2024 Feb 8;66:5. doi: 10.1186/s13028-024-00725-1 (PMC10851586; doi:10.1186/s13028-024-00725-1)
Supplement: Supplementary file 2 — Supplementary Material 2 [file 13028_2024_725_MOESM2_ESM.docx]

## **Supplementary material**

**Table S3:** Time between MRI scan and interview

| Time between MRI scan and interview | Number of dogs  (n=89) |
| --- | --- |
| 0-5 months | 13 |
| 5-12 months | 12 |
| 1-2 years | 14 |
| 2-3 years | 8 |
| 3-4 years | 10 |
| 4-5 years | 6 |
| 5-6 years | 5 |
| 6-7 years | 21 |

n, total number of dogs
