# Supplementary material for: Clinical predictors of syringomyelia in Cavalier King Charles Spaniels with chiari-like malformation based on owners’ observations
Source: Acta Vet Scand. 2024 Feb 8;66:5. doi: 10.1186/s13028-024-00725-1 (PMC10851586; doi:10.1186/s13028-024-00725-1)
Supplement: Supplementary file 3 — Supplementary Material 3 [file 13028_2024_725_MOESM3_ESM.docx]

## **Supplementary material**

## **Table S2:** Abnormal clinical and neurological findings in dogs examined in the University Hospital Neurology Specialist Clinic

| Clinical signs | CM and no SM  (n=7) | CM and SM 2.00-3.99 mm (n=11) | CM and SM ≥4.00 mm  (n=29) |
| --- | --- | --- | --- |
| Scratching neck or shoulder | 2 | 5 | 14 |
| Signs of neck pain | 3 | 5 | 9 |
| Pain from other regions | - | 2 | 9 |
| Strabismus | - | 3 | 5 |
| Ataxia | - | 1 | 6 |
| Head tilt | - | 1 | 6 |
| Dermatological findings (e.g. hyperemic skin, alopecia, otitis externa) | 1 | 2 | 4 |
| Aversion towards wearing collar/harness | 1 | 1 | 3 |
| Reduced menace response | - | - | 5 |
| Reduced palpebral reflex | - | - | 4 |
| Spontaneous scratching | 1 | - | 2 |
| Reduced proprioception | 1 | - | 2 |

Out of the 65 dogs examined at the University Hospital Neurology Specialist Clinic, 47 dogs had one or more abnormal clinical or neurological findings.
