# Supplementary material for: Clinical predictors of syringomyelia in Cavalier King Charles Spaniels with chiari-like malformation based on owners’ observations
Source: Acta Vet Scand. 2024 Feb 8;66:5. doi: 10.1186/s13028-024-00725-1 (PMC10851586; doi:10.1186/s13028-024-00725-1)
Supplement: Supplementary file 4 — Supplementary Material 4 [file 13028_2024_725_MOESM4_ESM.docx]

## **Supplementary material**

## **Table S4:** The relationship between Effusive Otitis Media (OME) and scratching of the neck or shoulder.

| Question | n | Answer | No OME | Unilateral OME | Bilateral OME | P |
| --- | --- | --- | --- | --- | --- | --- |
| Scratching neck or shoulder ^Q3^ | 88* | Yes/No | 31/20 | 10/8 | 11/8 | 0.92 |

* One dog was excluded, as its MRI scan did not include the inner ears.

OME, Effusive Otitis Media; n, total number of dogs, Q, question number referring to the questionnaire.
